# Development of an approach to monitor the manufacturing consistency of HIV rapid diagnostic tests: Panel qualification and potential impact on country programs

**DOI:** 10.1371/journal.pone.0284175

**Published:** 2023-04-10

**Authors:** David Jenkins, Roger Peck, Ashini Fernando

**Affiliations:** 1 Product Quality and Compliance, Durham, NC, United States of America; 2 Diagnostics Program, PATH, Seattle, WA, United States of America; University of Health and Allied Sciences, GHANA

## Abstract

Although regulatory bodies have standards that manufacturers of rapid diagnostic tests (RDTs) must meet for market approval, RDTs have no specific sampling and testing standards to monitor ongoing lot production, unlike pharmaceuticals and certain devices. With the importance of accurate diagnosis for improved health outcomes, independent quality assurance testing is key to ensuring the availability of high-quality RDTs, particularly in low-resource settings. This work develops an approach for HIV RDT lot testing, involving qualification of specimens to enable testing across various RDTs (namely Determine HIV-1/2, OraQuick HIV-1/2, Bioline HIV-1/2 3.0, UniGold HIV, and HIV Ag/Ab Combo). A sampling plan and acceptance criteria were developed per lot (approximating sensitivity and specificity) based on ISO 2859–1: 1999, using the test line response to a qualified panel (disease-positive and negative specimens) as the attribute. Based on general performance of HIV RDTs, an average % defective tests allowed per lot (acceptance quality limit) of 0.65% within ISO 2859–1: 1999 was selected, where RDTs are tested with 80 positives (accept 1 / reject 2 defective results) and 80 negatives (accept 1 / reject 2 defective results) per lot. Panel qualification was conducted with 83 positive and 84 negative serum specimens to select specimens that consistently provided expected results when tested in quadruplicate with three lots per product. While all products yielded consistent results with at least 80 negative specimens, only 4 products did the same for positive specimens. With this approach, each of these 4 RDT products can be tested with the qualified 80-positive specimen panel, requiring the other product to be tested with 20 specimens in quadruplicate. Additionally, this approach was adapted to evaluate HIV antibody/antigen combination tests with Ag panel qualification using p24 samples. While panels were qualified to monitor ongoing lot consistency of HIV RDTs, this approach could be mimicked with other types of diagnostics for monitoring manufacturing consistency, field investigation, small-scale stability checks, and proficiency testing.

## Introduction

Rapid diagnostic tests (RDTs), also commonly referred to as rapid test kits (RTKs), for HIV diagnosis are vital to public health programs that commonly rely on multi-product algorithms, where a patient is tested with different products brands for confirmation [1–5]. Diagnostic accuracy is critical to ensure the resources for treatment are appropriately applied to all patients in need, thus emphasizing the importance for overall quality assurance of country programs [6] and for implementing improvements in existing programs [7]. Inaccurate or discordant results can occur for a variety of reasons. False positives can arise from cross-reactivity with other types of antibodies [8–12] and false negatives may result from patients that have not yet seroconverted or with children treated with highly active antiretroviral therapy (HAART) [13]. Although appropriate testing algorithms help to minimize the occurrence of inaccurate patient diagnosis [14, 15], mechanisms for monitoring product quality (consistency of manufacturing) of the HIV RDTs entering the program are also important to ensure that proper RDT performance is being sustained [6].

Variations in RDT performance (quality) can potentially arise from a variety of sources, namely innate variability from within device components and the manufacturing process (including raw material and reagent suppliers); environmental differences (e.g., temperature and / humidity) within controlled laboratory settings versus field settings, including transportation supply chain and storage; user training and experience; and variations within target populations. Control lines within each RDT provide an indicator of test validity based on sample flow and conjugate response, where some RDTs implement control lines indicative of specimen addition. However, since these RDTs are designed to be qualitative, there are no built-in mechanisms to determine if the RDTs meet the claimed detection thresholds or range. Therefore, variations in manufacturing can yield lot-to-lot variations that can compromise RDT quality that in turn impacts RDT performance mainly around detection limits. Especially where RDTs are designed to detect multiple analytes (e.g. HIV-1 antibodies directed to different antigens: gp41, gp 120, gp160, antibodies to HIV-1 and HIV-2, and HIV antibody and HIV antigen), compromises in performance can result in significant programmatic setbacks that are further compounded by supply chain issues. The current level of post-market RDT surveillance in low- and middle-income countries (LMICs) is weak and is limited to reporting performance concerns such as high percentages of invalid results [16, 17] or false negative results for certain HIV-1 subtypes [18]. Against this backdrop, primary RDT quality control procedures are implemented by the manufacturer in the production and lot release processes included in their good manufacturing processes and quality management systems. Considering that HIV RDTs belong to the highest-risk category of in vitro diagnostics (IVDs), the development of an independent quality testing mechanism is imperative because there are no international standards or guidance for ongoing monitoring of product quality for RDTs that provide testing approaches based on statistical sampling with accept / reject criteria.

In the same manner that various publicly available test procedures (with acceptance criteria) exist for pharmaceuticals [19–21] and medical devices [22–24], there is a need for the development of standardized test procedures for independent monitoring of product quality for HIV RDTs and other diagnostics. Such independence is important to provide added assurances to the procurement processes and provide support and resources to product inquiries or investigations. Although guidance is available for evaluating HIV RDTs for general performance [25, 26], specific and comprehensive testing approaches are not readily available that provide acceptance criteria for lot-to-lot consistency based on statistical sampling approaches involving qualified panel testing (where a panel is comprised of multiple specimens). The work described herein provides a more standardized approach for independently monitoring HIV RDTs for quality assurance with details on panel qualifications for five prominent WHO-prequalified products: Determine HIV-1/2, Bioline HIV 1/2 3.0, Uni-Gold HIV, OraQuick HIV-1/2 for Ab detection, and HIV Combo for antibody/ antigen (Ab/Ag) detection. Although these RDTs cover a combination of specimens encompassing serum, plasma, whole blood, or oral fluid, this work has focused on serum panels and addresses questions pertaining to what number of samples per lot to test, what criteria should be applied, how to qualify panels across multiple RDTs, and what are the potential applications of this approach generally for diagnostics.

## Materials and methods

### Equipment

Calibrated micro-pipettes used were Rainin pipet-lite or pipet-lite XLS L2, L20, and/or L200. Calibrated digital timers were VWR® Traceable® Four-Channel Alarm Timer with Clock Model 62344–641. Calibrated thermometers were VWR® Traceable® Econo Refrigerator Thermometer Model 36934–132. Digital camera (single lens reflex) Nikon D70 was fitted with AFS Nikor 18-70mm 1:3.5–4.6G ED lens. A light box (Finnhomy Professional Portable Photo Studio Photo Light Studio Photo Tent Light box Table Top Photography Shooting Tent Box Lighting Kit, 16” x 16” Cube) was used and the light output was measured by Sekonic Spectromaster C-800-U: CCT = 5599k, Ra = 83.9, Lux = 9050lx, x = 0.3302 and y = 0.3464. Specimen Storage Tubes (0.5mL polypropylene screw cap cryovial (Sarstedt)) were used and provided by the Fred Hutchinson Cancer Research Center, where long-term specimen inventories are maintained. White background for reading tests was standard white printer/copier paper.

### Determining the number of positives/ negatives specimens for qualification

The number of specimens required for testing HIV RDTs, with appropriate acceptance criteria, was developed through a review of ISO 2859–1:1999 [27]. ISO 2859–1:1999 provides different sampling plans as a function of variables such as lot size, acceptance quality limit, and inspection level. Lot size refers to the number of units manufactured in a production lot. Acceptance quality limit (AQL) represents the percent level of defects in a lot that are tolerated or allowed by the end user. Depending on the product, HIV RDTs can generally be considered to have specificities and sensitivities of ~99%, which could correspond to an AQL of approximately 1%. ISO 2859–1:1999 provides seven inspection levels that are divided into general (III, II, I) and special (S-4, S-3, S-2, and S-1) categories. Level II is considered the default level, where consideration of other options is a function of the level of discrimination needed and on sample size logistics (III is the highest / S-1 is the lowest). Each sampling plan provides accept/reject criteria that correspond to the maximum number of failures that are acceptable and the minimum number of failures that reject the lot, respectively. For each sampling plan, ISO 2859–1:1999 provides information that allows one to determine an operating characteristic curve, which is a plot of the likelihood of lot acceptance/rejection during testing as a function of true percent non-conformity of the product through continual manufacturing. The following discussion is focused on single sampling plans (for normal inspection); ISO 2859–1:1999 provides options (more advanced circumstances) for double sampling plans and reduced or tightened inspection.

Different sampling plans were compared across several variables, namely lot size, acceptance quality limit, and inspection level. For lot size, a range of 35,001–500,000 units is understood to cover relevant sizes for RDTs. Acceptance quality limits of 0.65, 0.40, 0.25, and 0.15 were considered to provide options that are under a 1% defect level (to maintain ~99% sensitivity/specificity). Inspection Levels II, I, S-4, and S-3 were reviewed to encompass the default II level and more practical levels (lower amounts) based on sample availability. Also, lower sampling options were considered due to the difficulty of obtaining and maintaining the appropriate positive/negative specimens. Additionally, this lot testing approach is in the context of being conducted on product that has ostensibly been approved and released by the manufacturer’s standard quality control procedures, thereby allowing this approach to have less rigidity than a strict interpretation of ISO 2859–1:1999 and allowing the same approach to be broadly applied to RDTs, regardless of manufacturer, lot size, or product design (e.g., sensitivity or specificity).

**Fig 1:**
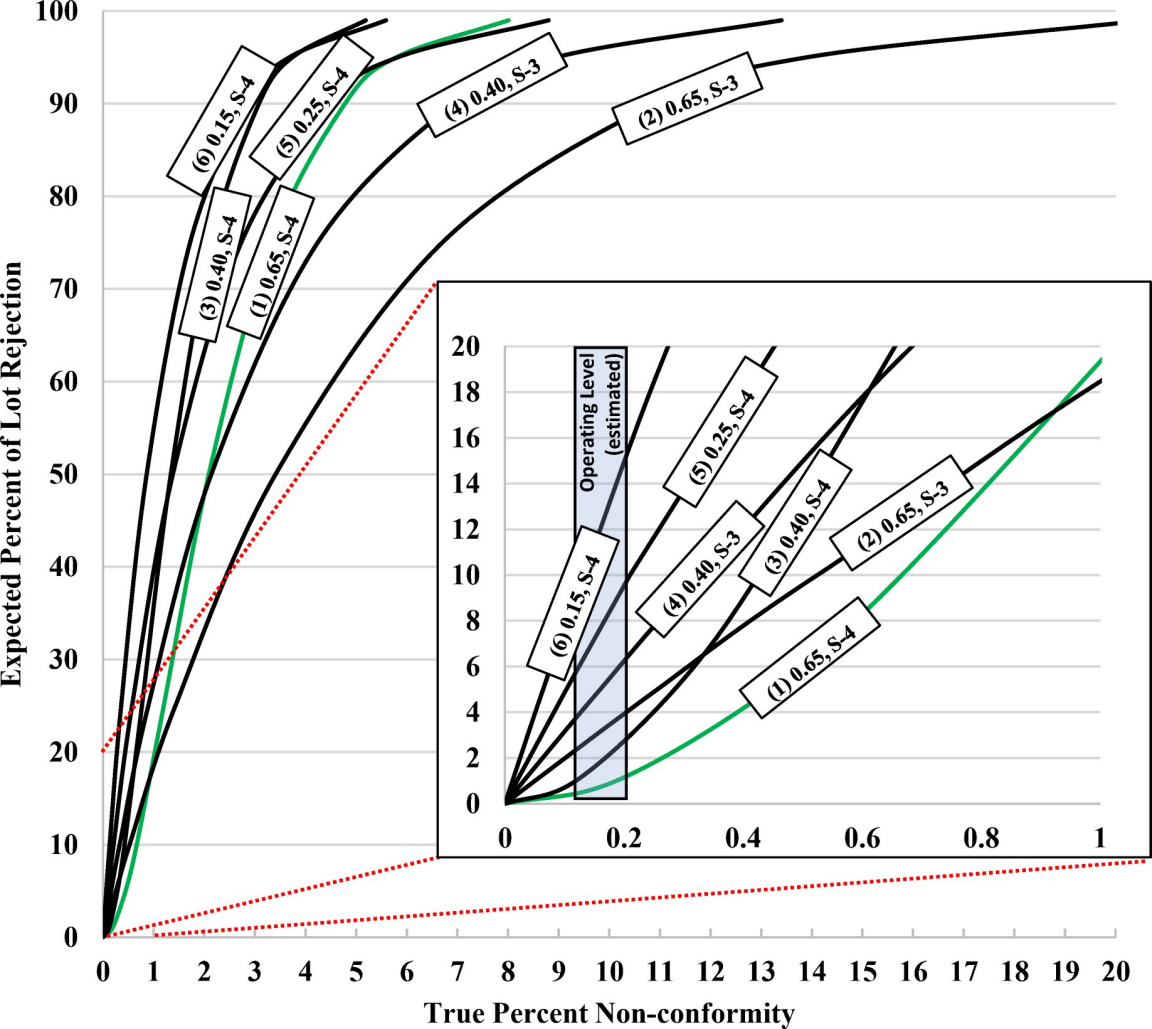
Operating characteristic curves from ISO 2859–1: 1999 for the lot size range of 35,001–500,000 for various sampling options (AQL, inspection level). For each AQL / inspection level provided in the figure, the respective number of samples and accept/reject ratio is listed as follows for single sampling plans at normal inspection: **(1)** 0.65, S-4, 80, 1/2 (green highlighted selected for use); **(2)** 0.65, S-3, 20, 0/1; **(3)** 0.40, S-4, 125, 1/2; **(4)** 0.40, S-3, 32, 0/1; **(5)** 0.25, S-4, 50, 0/1; **(6)** 0.15, S-4, 80, 0/1). Operating characteristic curve data in ISO-2859-1: 1999 is provided as expected percentage of lots to be accepted (%A) but presented here as an expected percentage of lots rejected (%R = 100 - %A) to visually correlate increases in percent non-conformity with increases in lot rejection.

Due to limited specimen availability, plans that utilize 200 samples or less for the entire lot size range were considered as viable options and are listed as follows (AQL, inspection level, number of specimens, accept / reject ratio): **(1)** 0.65, S-4, 80, 1/2; **(2)** 0.65, S-3, 20, 0/1; **(3)** 0.40, S-4, 125, 1/2; **(4)** 0.40, S-3, 32, 0/1; **(5)** 0.25, S-4, 50, 0/1; **(6)** 0.15, S-4, 80, 0/1). From ISO 2859–1:1999, operating characteristic curves for these respective options were plotted (Fig 1) at a 20% (full scale) and 1% (magnified scale) true percent non-conformity level. From these curves, any of the plans would perform well with catastrophic product failure because all would reject at least 80% of the lots at greater than 10% non-conformity. However, HIV RDTs were performing at an overall approximate non-conformity level of ~0.1–0.2% (percent of discordant RDT results with applied positive and negative specimens) based on initial data from the United States Agency for International Development’s Global Health Supply Chain Quality Assurance program (implemented by FHI 360). Therefore, sampling plan 2 (0.65, S-3) is not recommended for HIV RDTs because it is the least stringent at a 2–3% or greater non-conformity level. Sampling plans 4 (0.40, S-3), 5 (0.25, S-4), and 6 (0.15, S-4) are not recommended either because 5% or more of lots are expected to be rejected at a 0.1–0.2% non-conformity level (too stringent). Therefore, plan 1 (0.65, S-4) was chosen as the optimal plan for HIV RDTs. Relative to plan 3 (0.40, S-4), plan 1 (0.65, S-4) would reject fewer lots at the 0.1% - 0.2% non-conformity level while also requiring 45 fewer samples to conduct the evaluation.

The selection of plan 1 (0.65, S-4) would require 80 positive specimens and 80 negative specimens per lot of HIV RDTs evaluated. The more ideal approach is to test each lot with 80 unique specimens in single (both for positives and negatives), however, an alternate approach could be to test with 20 specimens in quadruplicate (for both positives and negatives) so that 80 total RDTs are tested each with positive and negative specimens. While the former approach is preferred in terms of maximum specimen diversity, the latter may be necessary depending on specimen cost and availability. This sampling and testing scheme (utilizing an AQL of 0.65) could be considered consistent with a product providing an overall sensitivity and specificity each of approximately 99.35% (or higher). From a review of operating characteristic curves (see Fig 1) [27], this evaluation scheme can be expected to screen out over 90% of the lots that exhibit true sensitivity and/or specificity of 95% or lower. Furthermore, there is a high probability (>80%) that lots will be found acceptable when operating at a true sensitivity and/or specificity of 99% or higher. For this evaluation scheme, the lower bounds on specificity / sensitivity when testing one lot are estimated in Table 1 [28–30] for the respective number of defects and confidence levels for testing scenarios involving either 80 specimens with 1 replicate each or 20 specimens with 4 replicates each. The higher level of the lower bounds for 80 unique specimens (1 replicate) indicates a closer correlation for results per lot with generally expected sensitivity/specificity levels over time relative to using 20 specimens (4 replicates), but the latter option is considered viable depending on the circumstances.

**Table 1:** Lower bounds on specificity and sensitivity, estimated through the Wilson scores approach [28–30], for the respective number of defects and confidence levels for testing scenarios involving either 80 specimens (with 1 replicate each) or 20 specimens (with 4 replicates each).

**80 specimens, 1 replicate** **Lower Bounds on Sensitivity / Specificity (n = 80, AQL = 0.65)**		
**Confidence Level**	**1 Defect**	**0 Defects**
95%	93.3%	95.4%
90%	94.6%	96.7%
80%	95.9%	98.0%
**20 specimens, 4 replicates** **Lower Bounds on Sensitivity / Specificity (n = 80, AQL = 0.65)**		
**Confidence Level**	**1 Defect**	**0 Defects**
95%	90.1%	92.2%
90%	92.2%	94.4%
80%	94.4%	96.5%

### Specimen sources

Human serum specimens were purchased from ProMedDx, LLC with available characterizations for individual positive and negative samples provided in respective certificates (i.e., serialized identifier, gender, age, and available test results). The specimens are collected from US donors and thus the positive specimens are not necessarily representative of the global genetic diversity of HIV and the negative specimens are not necessarily representative of the intended RDT target population. Donor age, gender, and race were not considered for inclusion in the specimen set. It is highly probable that due to the demographics of the positive specimen donor population (previously diagnosed HIV positive) that most or all donors may actively be on HIV antiviral treatment and are not necessarily representative of the intended RDT target population. As a contingency for an unknown level of unexpected results, and to meet the ideal ISO sampling plan of 80 specimens, more than 80 positives and 80 negatives were selected for the qualification. For HIV-1 positive serum evaluated through ProMedDx, 83 unique samples (30 mL of each in 3 separate 10 mL vials) were selected based on the highest volumes (mL) per sample available and on “Reactive” results for qualitative tests (Abbott EIA and PRISM ChLia) or positive indicating numerical results for qualitative tests (Abbott Architect and Siemens Centaur). All positive samples were selected with the above criteria and with also having HIV viral load data provided in the ProMedDx certificate from the Roche PCR test, tested “Reactive” on the Abbott HIV AB HIV-1/HIV-2 (rDNA) EIA, and tested “Non-Reactive” for HBsAg and HCV on Abbott PRISM (ChLIA). For negative serum, 84 unique samples (~175 mL of each in a single bulk container) were purchased with certified information with “Non-Reactive” results with PRISM ChLia (Viral testing for HIV, HBsAg, HCV), “Non-Reactive” results with Roche Cobas TaqScreen (NAT Testing for HIV, HBV, HCV), and “Non-Reactive” results for RPR (syphilis) with the Arlington Scientific Card Test. All samples from ProMedDx were additionally labeled with a simplified code (correlated with the serialized identifier from ProMedDx within internal database). Positives were coded as P001PM through P083PM and negatives were coded as N001PM through N084PM. The WHO International Standard HIV-1 p24 Antigen NIBSC code: 90/636 was used as the antigen positive (Ag) sample and diluted in negative serum.

### HIV RDTs for panel qualification

Various products were obtained from the respective manufacturers for use in panel qualification. Approximately 1000 units from each of 3 lots were obtained for use from Determine HIV-1/2 (Alere, Product number: 7D23), HIV Combo (Alere, Product number: 7D2843), Uni-Gold HIV (Trinity Biotech, Product number: 1206502), SD Bioline HIV-1/2 3.0 (Standard Diagnostics, Product number: 03K10, 03FK16), and OraQuick HIV-1/2 (OraSure, Product number: 5X4-0010). Product names will not be provided in correlation with the Ab results but will rather be referenced with a product code to protect manufacturer confidentiality. HIV Combo will be directly referenced when discussing Ag results because it was the only product utilized that could detect Ag.

### Panel qualification

Because long-term storage conditions are required at -80°C (approximately -70°C to -90°C), sample specimens were obtained from the respective suppliers in bulk containers that were received frozen (packed in dry ice). Specimens need to be thawed to room temperature for actual testing of RDTs, however frequent freeze-thaw cycles will degrade specimen integrity. Therefore (after receipt from the supplier), a portion of each specimen was thawed at room temperature and aliquoted to smaller volumes for long-term storage at -80°C so that only the volume needed for testing can be removed for use to incorporate a single thaw cycle. Positives already came in 3 separate 10 mL bulk containers and only one container was thawed for further aliquoting. Negative specimens came in ~175 mL units that were thawed and frozen in multiple 10 mL containers, where only one 10 mL container per specimen was aliquoted and used for qualification. For this panel qualification, an optimal aliquot level was determined to yield minimal wastage of specimen volume over time based on manufacturer instructions outlining the volume required for testing and on the number of lots that can be tested. The following volumes of plasma/serum are needed for a single analysis with each of the listed HIV RDTs (Determine—50 μl [31, 32]; Uni-Gold–one drop (~ 50 μl) [33]; SD Bioline—10 μl [34]; OraQuick—~5+ μl [35]). Various aliquot amounts were considered, but the 250 μl aliquoting volume was selected based on input from industry representatives to minimize impact from potential volume loss over time.

For specimen aliquoting, the commercially sourced sample (10 mL container) was thawed at 23°C or cooler (without the use of a heating block). The thawed sample was adequately mixed (i.e., via vortexing or inversion over 10 times) and then aliquoted into separate 250 μl portions contained in specimen storage tubes. Each separate specimen aliquot was given a label that links or correlates the source code provided by the commercial source. All specimen aliquots were returned to appropriate freezer conditions until ready for use. It should be noted that each 250 μl aliquot is only to be used as a single thaw sample but can be used multiple times if stored at 2–8°C for 5 days.

For each lot of RDTs received, 4 replicates of each specimen (positive and negative) were conducted, thus yielding 12 replicates across each RDT type per specimen. The replicates establish performance consistency from test-to-test and lot-to-lot. All testing was conducted according to the section outlining the standard procedure below with adaptations to specific manufacturer’s instructions for use for each product. The implementation of four replicates per lot/specimen combination is conducted to allow flexibility in using the panel for under 80 specimens / single application or 20 specimens / 4 replicate approach. A given specimen and lot are considered qualified provided 100% concordance is obtained from the results, meaning no false positives or false negatives (also screening for invalid tests) are observed. Qualified lots can subsequently be used as reference (control) lots during routine testing.

When the test was read at the required time, a qualitative intensity was noted for the test line with one of the following options: 0 –negative; w–test line is faintly observable; 1 –test line intensity is weaker than the control line; 2 –test line intensity is comparable to the control line; 3—test line intensity is stronger than the control line. Two technicians independently interpreted and recorded the test results. In the case of discrepant quantitative results, a third independent reader interpreted the tests. For information and general documentation purposes, digital images were obtained for test RDTs between the minimum / maximum read time (essentially within several minutes of when the visual judgement is made for the set of samples). Digital images were taken at the time of testing.

### Standardized procedure to evaluate HIV RDTs with qualified panels

For each RDT production lot to be evaluated, a data collection form is prepared that indicates the specific panel to be used for the testing, where the specimens identified are thawed (at room temperature without use of heating block) for use that day. The remaining specimen is not refrozen for later use but may be stored at 2–8°C for 5 days for future use. A total of 80 HIV positive specimens (81 if a combination Ab/Ag test is being evaluated) and 80 HIV negative specimens are used to evaluate each production lot. Specimen thawing should be coordinated with the number of lots that could readily be completed in a single day so that the proper set of panels are thawed and used that day. RDTs should be placed on the laboratory bench top that is covered with a white background (i.e., white laboratory paper towels or bench pads) to provide a uniform background for result interpretation. A maximum of 10 RDTs are to have specimens applied at the same time. Each RDT (unit) is labeled to indicate the specimen that will be used for the evaluation of that specific unit, referencing the data collection form for lot under evaluation. RDTs shall be tested according to the package insert/instructions for use (IFU). Specific items of note are the following: the volume of specimen and method for specimen application, volume of kit buffer, and minimum / maximum reading time after specimen application. Deviations from the package insert/IFU must be recorded and explained.

Each specimen should be thoroughly mixed (e.g., vortexed for approximately 2 seconds) and subsequently applied to the test device at the specified volume. If the product instructions allow for specimen addition via a specimen transfer device not included in the kit, specimen addition using a calibrated micropipette is preferred. If the procedure calls for the addition of a buffer, the buffer is applied according to the package insert. A timer is started after the addition of specimen or buffer (if used) to the first RDT (of the group selected for evaluation during the same time period). A single timer can be used for the evaluation of a set of devices (maximum of 10 at a time); the use of multiple timers allows for parallel evaluation of multiple sets of devices. The laboratory environment should use general overhead lighting and not use directed incandescent lighting to prevent inadvertent heating of samples/kits during testing. Ideal lighting would occur from general overhead fluorescent or LED light at a range of approximately 2000–3000 Lux. Results are read during the specified time period indicated in the package insert. The time of reading (relative to the sample application time) for each subset of tests evaluated at the same time should be recorded. All results should be interpreted according to package insert (i.e., positive/+/reactive, negative/-/non-reactive, inconclusive/indeterminate, invalid, etc.) and recorded on the data collection form (with photographs, if feasible). Unexpected results (e.g., weak positives, false positives, false negatives, other anomalies, etc.) should be recorded in the data sheet and photographed for records.

From the data collection forms, results should be compiled noting the test results and agreement (conforming or nonconforming) with the expected results for each specimen. The number of conforming results for the panel shall be tallied and recorded. The number of invalid tests should be recorded as well (less than or equal to 1% invalid tests could be considered acceptable). Based on ISO 2859–1:1999 (Single Sampling, Normal Inspection, S-4 Level, Sample Size Code J, AQL 0.65), no more than one nonconforming RDT result out of 80 RDT results is acceptable (for each set of positive and negative panels). If no more than one nonconforming test result is observed for each of the positive (associated with sensitivity) and the negative panels (associated with specificity), the lot under evaluation is considered acceptable.

If 2 or more nonconforming test results are found for either the positive or negative panels, the results are considered unacceptable, and a laboratory investigation needs to be completed, which reviews and documents the following items to assess whether any source of laboratory error can be found: review inventory forms, kit storage temperature and humidity check, kit testing temperature and humidity check, specimen storage check, kit packaging check, kit expiry date check, kit procedure/ instructions/ insert check, review of calculations, review of equipment calibration status used for testing, review of data collection forms, review of correspondence, review all kit paperwork, photographs of nonconforming results, volume of remaining specimens(s), discordant tests retained, repeat testing of kit with new specimen aliquots, testing of specimen with reference lots. A reference lot is defined as a lot that was previously tested that provided no nonconformities for either positive or negative samples and still within product shelf life.

If a laboratory error is found to be the root cause of the unexpected result, the original data shall be maintained on file for the lot and testing repeated after appropriate corrective / preventative actions are implemented. If no laboratory error is found, a report should be prepared that includes the out of specification investigation summary and the testing details from the investigation.

## Results

During panel qualification, although there were certain specimens that yielded unexpected results, there was generally a high level of concordance with the specimens and the expected responses for all five product types. Panel qualification results are summarized in Table 2 for each of the HIV RDT products evaluated (provided in more detail in S1 Table), where Table 3 consolidates the results for specimens that specifically yielded discordant results with visual intensities indicated.

**Table 2:** Panel qualification summary as a function of product type.

Product	Positives (Ab) (Qualified)		Positives (Ab) (Not Qualified)		Negatives (Ab) (Qualified)		Negatives (Ab) (Not Qualified)
A	81 Total P002PM-P041PM; P043PM-P083PM		2 Total P001PM (N); P042PM (N)		84 Total N001PM-N084PM		0 Total
B	81 Total P002PM-P073PM; P075PM-P083PM		2 Total P001PM (N); P074PM (N)		83 Total N001PM-N077PM; N079PM-N084PM		1 Total N078PM (P)
C	74 Total P002PM-P026PM; P030PM-P035PM; P037PM; P039PM-P041PM; P043PM-P064PM; P066PM-P073PM; P075PM-P083PM		9 Total P001PM, P042PM, P074PM (N); P028PM^a^, P065PM^b^ (partial N); P027PM^c^, P029PM^c^, P036PM^c^, P038PM^c^ (IV)		84 Total N001PM-N084PM		0 Total
D	80 Total P002PM-P041PM; P043PM-P073PM; P075PM-P083PM		3 Total P001PM (N); P042PM^d^, P074PM^d^ (N)		83 Total N001PM-N041PM; N043PM-N084PM		1 Total N042PM^e^ (P)
E	81 Total P002PM-P073PM; P075PM-P083PM		2 Total P001PM (N); P074PM (N)		83 Total N001PM-N045PM; N047PM-N084PM		1 Total N046PM (P)
HIV Combo	P24 Antigen Standard Results						
	For five frozen diluted p24 standards (diluted in N001PM), all dilutions at or above the stated assay limit of detection (2 IU/mL) were positive at both 20 and 40 minutes.						
	Specimen ID	Concentration		20 min result		40 min result	
	P002PA	50 IU/mL		Positive		Positive	
	P003PA	10 IU/mL		Positive		Positive	
	P004PA	5 IU/mL		Positive		Positive	
	P005PA	2 IU/mL		Positive		Positive	
	P006PA	1 IU/mL		Positive and Negative		Positive	
	P002PA, P004PA, and P005PA should be used for routine testing. For singlicate testing, specimens P002PA and P004PA must be positive, while P005PA should be positive (taken for information).						

Specimens considered qualified provided the expected result (positive or negative, respectively) on all replicates of all lots tested (n = 12).

N–negative reaction; P–positive reaction; partial N–partial negative; IV–invalid results

a—Specimen P028PM gave one false negative on the four replicates of two lots of the test

b—Specimen P065PM gave one false negative on the four replicates of one lot of the test

c–Specimens P027PM, P029PM, P036PM, and P038PM gave at least one invalid result on the four replicates in at least one of the lots tested. Invalid results appeared to be related to absorption problems with the specimens

d–Specimens P042PM, and P074PM gave negative/nonreactive results at 10 minutes, the earliest time point of the reading window, on one of the three lots tests used in the panel qualification

e—Specimen N042PM gave positive/reactive results at 20 minutes, the outer time point of the reading window, on two of the three lots used in the panel qualification

**Table 3:** Summary of positive and negative Ab specimens that provided discordant results for any product type.

Specimen Type*	Product A	Product B	Product C	Product D	Product E
P001PM	N (12)	N (12)	N (12)	N (12)	N (12)
P027PM	2	2	1.3 (11), IV (1)	3	1.7
P028PM	W	1	N (2), W (10)	1	1
P029PM	2.8	2	IV (12)	1.4	1
P036PM	3	2	2.5 (2), IV (10)	2.3	2
P038PM	3	2	1.3 (8), IV (4)	1	2
P042PM	N (12)	W	N (12)	N (4), W (8)	W
P065PM	1	1	1 (11), N (1)	1.3	1
P074PM	1	N (12)	N (12)	N (4), W (8)	N (12)
N042PM	N	N	N	N (4), W (8)	N
N046PM	N	N	N	N	W (4), 1 (8)
N078PM	N	W (12)	N	N	N

Numerical values provide the average result for the scale of 1–3, where N and/or W are indicated as appropriate. IV indicates invalid results. Some specimens provided mixed results across the replicates and are noted with multiple indicators. For yellow and red cells, values in parentheses indicate the number of RDT units out 12 that yielded the specific result. Results provided in green cells are across 12 RDTs.

*Green cells = expected results, Yellow cells = include invalid results with any of the 12 RDTs during qualification, Red cells = include unexpected results with any of the 12 RDTs during qualification.

Focusing on panels for Ab detection, all products were able to qualify at least 83 of the 84 negative specimens used in the evaluation. Across the products evaluated, none of the negative specimen yielded false-positive results across multiple products. False positive results were obtained on the following product / negative specimen combinations: RDT B–N078PM; RDT D–N042PM; RDT E–N046PM.

However, several positive specimens yielded negative results across several different RDTs. P001PM yielded negative results across all five brands, where the likely cause could be differences between the limit of detection of RDTs when compared to detection assays used by supplier (ProMedDx). P042PM and P074PM were discordant (negative) for most RDT types. RDTs A, C, and D yielded negative results with P042PM, while RDTs B, C, D, and E yielded negative results with P074PM. The RDT types that yielded an expected result with these discordant specimens demonstrated a weaker test line relative to the control line, indicating potentially low HIV antibody levels. Overall, RDTs A, B, D, and E were all able to have at least 80 positive specimens qualified for Ab detection.

RDT C had 74 specimens qualified out of the 83 positive specimens. Of the unqualified positive specimens, three were unqualified across other brands as well: P001PM, P042PM, and P074PM. Of the remainder, P028PM and P065PM yielded two and one false negatives, respectively, within the twelve replicates used during the qualification. It is noteworthy that these two specimens yielded low line intensities across the other brands although none resulted in false negative responses. Positive specimens P027PM, P029PM, P036PM, and P038PM provided various levels of invalid results with RDT C apparently due to absorption and inconsistent flow problems during the qualification. The data provided by ProMedDx was reviewed for any reason for the trend (seemingly clustered set of invalids) and none could be discerned from any of the information available. This lower level of positive specimen qualification for RDT C resulted in the need to activate the use of 20 positive specimens with 4 replicates each to allow 80 RDTs to be tested per lot for this specific RDT only (which is an alternative option when RDTs don’t yield the expected results with at least 80 specimens).

A panel involving p24 antigen specimens were only applicable for HIV Combo. Application of p24 antigen at different concentrations yielded expected results per claimed limit of detection (2 IU/mL), with partial positivity at lower levels (1 IU/mL). For a variety of test line intensity results, specimens P002PA, P004PA, and P005PA should be used for routine lot testing of HIV Combo. Because of the higher predominance of Ab detecting HIV RDTs, the criteria used during routine testing of Ag detecting diagnostics will be much less statistically based where a single application of specimens P002PA and P004PA must be positive and P005PA should be positive.

## Discussion

HIV RDTs are procured in high volumes by a variety of donors for use in different public health programs. Because no international standards exist for monitoring product quality of HIV RDTs, this work provides an approach to establishing a statistically supported mechanism to test HIV RDTs against a standardized panel (reflective of RDT performance criteria) to independently monitor manufacturing consistency. This approach is not intended to replace the lot release testing first performed by the manufacturer with their own internal panels, provide a mechanism to verify the clinical studies originally conducted by the manufacturer to establish product sensitivity and specificity levels, nor provide verification of a given country’s algorithm for patient diagnosis.

Although inaccuracies (discordances) with HIV RDTs can originate from biological sources (i.e., cross reactivity with other antibodies or incomplete seroconversion [8–13]) that can be minimized with appropriate diagnostic testing algorithms [14, 15], inaccuracies could also result from laboratory sources [6] specifically pertaining to technique and sample handling. Various types of sample handling issues could potentially impact the accuracy and consistency of results, such as the type of anticoagulants added (for plasma specimens), improper specimen storage (long term storage at -80°C is not possible because of equipment limitations), specimen thawing is too rapid because additional heat is applied, multiple freeze thaw cycles, improper mixing before application to RDT (i.e., lack of vortexing), lack of white background to read results, and inadequate lighting or use of incandescent lighting that could inadvertently heat specimens. If discordant results are observed with this procedure where a production lot is deemed to be out of specification (OOS) to the applied criteria, a laboratory investigation should be conducted to review the following aspects: proper specimen handling, proper RDT storage, current staff training, correct raw data calculations, and appropriately calibrated and maintained supporting equipment.

Although the primary purpose of this work is to routinely monitor RDT quality, there are several other applications of the established set of panels and the implemented criteria. With the importance of external quality assurance and proficiency testing [36–41], the panels could be used to establish new smaller scale proficiency programs with other labs or be included within existing proficiency programs for enhanced training and laboratory quality control [42, 43]. The use of the digital image analysis may enhance training approaches where line intensities are observed from images of HIV RDTs [44, 45]. The RDT lots used during the panel qualifications could also be used as “control lots” for additional information (i.e., consistency of specimen responses) during an OOS investigation. Furthermore, the panels and control lots could be used to assist in investigations that may arise from field complaints/evaluations [46]. For the purposes of additional information (not to replace required stability studies conducted by manufacturers), the panels may also be used to evaluate HIV RDTs exposed to adverse environmental conditions during small-scale stability studies [47–49] or to conduct programmatic verification of product performance of locally warehoused HIV RDTs.

The panel qualification process discloses the materials used and the approach implemented to provide transparency on the evaluation process for other groups to replicate, as appropriate. For this work, volumes of each panel were purchased so that a relatively large number of lots of HIV RDTs could be tested from a single panel qualification, while simultaneously allowing enough material to share with manufacturers or other laboratories for further investigation in the event of unexpected (discordant) results. With continued collaboration with appropriate stakeholders (manufacturers and testing laboratories), this approach could be further improved for robustness and harmonization to properly establish a standardized test method for monitoring product quality of HIV RDTs. Areas of future research are intended to expand HIV diversity, including HIV-2 and HIV-1 clade variants, and HIV-1 specimen dilutions for more comprehensive testing, if needed. This approach is not only intended to have similarities to approaches already available for other medical devices and pharmaceuticals, but it could readily be applied to other high-risk diagnostics such as syphilis, HIV/syphilis combo, hepatitis B or C, malaria and cryptococcus.

### Conclusions

Due to a lack of publicly available international standards for quality assurance of diagnostic devices, an approach was developed for monitoring consistency of manufacturing for HIV RDTs based on ISO 2859–1:1999 sampling plans and assessment criteria. From a review of ISO 2859–1:1999 and performance characteristics for HIV RDTs, an acceptance quality limit (AQL) of 0.65 was selected for application towards HIV serology/ab detecting RDTs. Based on a 0.65 AQL, the following criteria for each lot were developed: 80 HIV RTKs tested with Ab positive specimens (accept 1 defective result / reject 2 defective results) and 80 HIV RTKs tested Ab negative specimens (accept 1 defective result / reject 2 defective results). To perform this type of evaluation, Ab positive and negative specimens (panels) needed to be qualified to establish a baseline response per product type for future use in monitoring ongoing product consistency. Five prominent HIV RDTs brands were used in the panel qualification. For negative panels, 80 unique negative specimens were qualified for all RDT brands. For positive panels, 80 unique positive specimens were qualified for brands A, B, D, and E. Product C was only able to have 74 positives qualified, resulting in future monitoring with 20 qualified positives with 4 RDTs tested per specimen. Using p24 spiked samples, panel qualification was also conducted for Ag detecting RTKs (HIV Combo), where an approach was provided for ongoing QC monitoring for 4th generation antigen/antibody combination products. This qualification activity has provided a mechanism for monitoring HIV RDTs and could readily be replicated in a similar manner with other types of diagnostics. Furthermore, this set of panels and basic testing approach may be useful for field investigation, small scale stability checks, and proficiency testing amongst multiple lab sites.

## Supporting information

S1 Table Summary of positive and negative Ab specimens that were qualified (Yes/Green) and not qualified (No/Red) by RDT product. (DOCX)

## References

[1] Kaleebu P, Kitandwe PK, Lutalo T, Kigozi A, Watera C, Nanteza MB, et al. Evaluation of HIV-1 rapid tests and identification of alternative testing algorithms for use in Uganda. BMC Infectious Diseases. 2018; 18: 93. doi: 10.1186/s12879-018-3001-4 29482500 PMC6389083

[2] Nguyen VTT, Best S, Pham HT, Troung TXL, Hoang TTH, Wilson K, et al. HIV point of care diagnosis: preventing misdiagnosis experience from a pilot of rapid test algorithm implementation in selected communes in Vietnam. Journal of the International AIDS Society. 2017; 20(6): 21752. doi: 10.7448/IAS.20.7.21752 28872279 PMC5625549

[3] Shanks L, Klarkowski D, O’Brien DP. False Positive HIV Diagnoses in Resource Limited Settings: Operational Lessons Learned for HIV Programmes. PLOS ONE. 2013; 8(3): e59906. doi: 10.1371/journal.pone.0059906 23527284 PMC3603939

[4] Shanks L, Siddiqui MR, Kliescikova J, Pearce N, Ariti C, Muluneh L, et al. Evaluation of HIV testing algorithms in Ethiopia: the role of the tie-breaker algorithm and weakly reacting test lines in contributing to a high rate of false positive HIV diagnoses. BMC Infectious Diseases. 2015; 15: 39. doi: 10.1186/s12879-015-0769-3 25645240 PMC4331460

[5] Shodell D, Nelson R, MacKellar D, et al. Low and Decreasing Prevalence and Rate of False Positive HIV Diagnosis—Chókwè District, Mozambique, 2014–2017. MMWR Morb Mortal Wkly Rep. 2018; 67: 1363–1368. 10.15585/mmwr.mm6749a3 30543600 PMC6300074

[6] Parekh BS, Kalou MB, Alemnji G, Ou CY, Gershy-Damet GM, Nkengasong JN. Scaling Up HIV Rapid Testing in Developing Countries Comprehensive Approach for Implementing Quality Assurance. American Journal of Clinical Pathology. 2010; 134(4): 573–584. doi: 10.1309/AJCPTDIMFR00IKYX 20855638

[7] Smallwood M, Pai NP. Improving the Quality of Diagnostic Studies Evaluating Point of Care Tests for Acute HIV Infections: Problems and Recommendations. Diagnostics. 2017; 7(1): 13. doi: 10.3390/diagnostics7010013 28273857 PMC5373022

[8] Klarkowski D, Glass K, O’Brien D, Lokuge K, Piriou E, Shanks L. Variation in Specificity of HIV Rapid Diagnostic Tests over Place and Time: An Analysis of Discordancy Data Using a Bayesian Approach. PLOS ONE. 2013; 8(11): e81656. doi: 10.1371/journal.pone.0081656 24282615 PMC3840056

[9] Kosack CS, Page AL, Beelaert G, Benson T, Savane A, Ng’ang’a A, et al. Towards more accurate HIV testing in sub-Saharan Africa: a multi-site evaluation of HIV RDTs and risk factors for false positives. Journal of the International AIDS Society. 2017; 20:21345. 10.7448/IAS.20.1.21345 PMC5467586 28364560

[10] Kosack CS, Shanks L, Beelaert G, Benson T, Savane A, Ng’ang’a A, et al. HIV misdiagnosis in sub-Saharan Africa: performance of diagnostic algorithms at six testing sites. Journal of the International AIDS Society. 2017; 20: 21419. doi: 10.7448/IAS.20.1.21419 28691437 PMC5515032

[11] Shanks L, Ritmeijer K, Piriou E, Siddiqui MR, Kliescikova J, Pearce N, et al. Accounting for False Positive HIV Tests: Is Visceral Leishmaniasis Responsible? PLOS ONE. 2015; 10(7): e0132422. doi: 10.1371/journal.pone.0132422 26161864 PMC4498794

[12] Shanks L, Siddiqui MR, Abebe A, Piriou E, Pearce N, Ariti C, et al. Dilution testing using rapid diagnostic tests in a HIV diagnostic algorithm: a novel alternative for confirmation testing in resource limited settings. Virology Journal. 2015; 12: 75. doi: 10.1186/s12985-015-0306-4 25972188 PMC4432962

[13] Yogev R. Rapid HIV testing for developing countries: The challenge of false-negative tests. Proc. Of SPIE. 2012; 8371: Sensing Technologies for Global Health, Military Medicine, Disaster Response, and Environmental Monitoring II; and Biometric Technology for Human Identification IX, 83710C–1. 10.1117/12.924574

[14] Kravitz Del Solar AS, Parekh B, Douglas MO, Edgil D, Kuritsky J, et al. A Commitment to HIV Diagnostic Accuracy a comment on "Towards more accurate HIV testing in sub-Saharan Africa: a multi-site evaluation of HIV RDTs and risk factors for false positives ’and’ HIV misdiagnosis in sub-Saharan Africa: a performance of diagnostic algorithms at six testing sites" Journal of the International AIDS Society. 2018; 21(8): e25177. doi: 10.1002/jia2.25177 30168275 PMC6117497

[15] Kufa T, Kharsany ABM, Cawood C, Khanyile D, Lewis L, Grobler A, et al. Misdiagnosis of HIV infection during a South African community-based survey: implications for rapid HIV testing. Journal of the International AIDS Society. 2017; 20(6): 21753. doi: 10.7448/IAS.20.7.21753 28872274 PMC5625550

[16] World Health Organization. Field Safety Notice DLT/FSN.001. 2011. Available from: https://www.who.int/diagnostics_laboratory/procurement/111201_productalert_for_product0027_mx012.pdf (Accessed 4 March 2019).

[17] World Health Organization. Updated Field Safety Notice DLT/FSN.001. 2012. Available from: http://www.who.int/diagnostics_laboratory/procurement/120106_final_update_info_sd_bioline_hiv_rtd.pdf (Accessed 4 March 2019).

[18] Alere Medical Co. Urgent Field Safety Notice. 2014. Available from: https://laegemiddelstyrelsen.dk/da/udstyr/sikkerhedsmeddelelser/2014/alere-determine-hiv-12-agab-combo/~/media/A6ABA990A40E46729890FC6680846D0F.ashx (Accessed 8 June 2022).

[19] United States Pharmacopeia and National Formulary (USPNF). Rockville, MD: United States Pharmacopeia Convention; 2022.

[20] British Pharmacopeia (BP). London, UK: British Pharmacopeia Commission Office; 2022.

[21] The International Pharmacopeia. Geneva, Switzerland: World Health Organization; Tenth Edition. 2020. Available from: http://apps.who.int/phint/en/p/docf/ (Accessed 8 June 2022).

[22] ISO 4074:2015 Natural rubber latex male condoms–Requirements and test methods. ISO Copyright Office. Geneva: International Organization for Standardization; 2015.

[23] ISO 25841:2017 Female condoms–Requirements and test methods. ISO Copyright Office. Geneva: International Organization for Standardization; 2017.

[24] ISO 7439:2015 Copper-bearing contraceptive intrauterine devices–Requirements and tests. ISO Copyright Office. Geneva: International Organization for Standardization; 2015.

[25] World Health Organization. WHO/HIV/2015.27. Annex 8: Ensuring the quality of HIV testing services. 2015. Available from: http://apps.who.int/iris/bitstream/10665/180227/1/WHO_HIV_2015.27_eng.pdf (Accessed 8 June 2022).

[26] World Health Organization. Post-Market Surveillance of In Vitro Diagnostics. 2015. Available from: http://apps.who.int/iris/bitstream/10665/255576/1/9789241509213-eng.pdf?ua=1 (Accessed 8 June 2022).

[27] ISO 2859–1:1999 Sampling procedures for inspection by attributes–Part 1: Sampling schemes indexed by acceptance quality limit (AQL) for lot-by-lot inspection. ISO Copyright Office. Geneva: International Organization for Standardization; 1999.

[28] Newcombe RG. Two-Sided Confidence Intervals for the Single Proportion: Comparison of Seven Methods. Statistics in Medicine. 1998; 17: 857–872. 10.1002/(SICI)1097-0258(19980430)17:8<857::AID-SIM777>3.0.CO;2-E 9595616

[29] Saha KK, Miller D, Wang S. A Comparison of Some Approximate Confidence Intervals for a Single Proportion for Clustered Binary Outcome Data. International Journal of Biostatistics. 2016; 20150024. doi: 10.1515/ijb-2015-0024 26569139

[30] Gulliford MC, Adams G, Ukoumunne OC, Latinovic R, Chinn S, Campbell MJ. Intraclass correlation coefficient and outcome prevalence are associated in clustered binary data. Journal of Clinical Epidemiology. 2005; 58: 246–251. doi: 10.1016/j.jclinepi.2004.08.012 15718113

[31] Determine HIV-1/2 Ag/Ab Combo (Abbott). Instructions for Use. 2021. Available from: https://www.fda.gov/downloads/BiologicsBloodVaccines/BloodBloodProducts/ApprovedProducts/PremarketApprovalsPMAs/UCM364698.pdf (Accessed 10 June 2022).

[32] WHO Prequalification of Diagnostics Programme Public Report. Determine HIV-1/2. PQDx 0033-013-00. 2022. Available from: https://extranet.who.int/pqweb/sites/default/files/PQDx_0033-013-00_DetermineHIV_1-2_v9.0.pdf (Accessed 10 June 2022).

[33] Uni-Gold HIV1/2. UNICEF Supply Catalogue. 2018. Available from: https://supply.unicef.org/s0003406.html (Accessed 10 February 2023).

[34] WHO Prequalification of Diagnostics Programme Public Report. Bioline HIV-1/2 3.0. PQDx 0027-012-00. 2020. Available from: https://extranet.who.int/pqweb/sites/default/files/PQDx_0027-012-00_BiolineHIV_1-2-3_v5.pdf (Accessed 10 June 2022).

[35] OraQuick HIV1/2. UNICEF Supply Catalogue. 2018. Available from: https://supply.unicef.org/s0003416.html. (Accessed 10 February 2023).

[36] Bello-Lopez JM, Castaneda-Garcia C, Munoz-Estrada C, Machorro-Perez AJ. External quality control program in screening for infectious diseases at blood banks in Mexico. Transfusion and Apheresis. 2018; 57(1): 97–101. doi: 10.1016/j.transci.2018.01.004 29452838

[37] Jean Louis F, Excellent ML, Anselme R, Buteau J, Stanislas M, Boncy J, et al. External quality assessment for HIV rapid tests: challenges and opportunities in Haiti. BMJ Global Health. 2018; 3(6): e001074. doi: 10.1136/bmjgh-2018-001074 30498590 PMC6254742

[38] Johnson CC, Fonner V, Sands A, Ford N, Obermeyer CM, Tsui S, et al. To err is human, to correct is public health: a systematic review examining poor quality testing and misdiagnosis of HIV status. Journal of the International AIDS Society. 2017; 20(6): 21755. 10.7448/IAS.20.7.21755 28872271 PMC5625583

[39] Kyaw LL, Nozaki I, Wada K, Oo KY, Tin HH, Yoshihara N. Ensuring accurate testing for human immunodeficiency virus in Myanmar. Bulletin of the World Health Organization. 2015; 93(1): 42–46. doi: 10.2471/BLT.14.138909 25558106 PMC4271681

[40] Mwangala S, Musonda KG, Monze M, Musukwa KK, Fylkesnes K. Accuracy in HIV Rapid Testing among Laboratory and Non-laboratory Personnel in Zambia: Observations from the National HIV Proficiency Testing System. PLOS ONE. 2016; 11(1): e0146700. doi: 10.1371/journal.pone.0146700 26745508 PMC4706302

[41] Wesolowski LG, Ethridge SF, Martin EG, Cadoff EM, MacKellar DA. Rapid Human Immunodeficiency Virus Test Quality Assurance Practices and Outcomes among Testing Sites Affiliated with 17 Public Health Departments. Journal of Clinical Microbiology. 2009; 47(10): 3333–3335. doi: 10.1128/JCM.01504-09 19692557 PMC2756945

[42] Jaya Z, Drain PK, Mashamba-Thompson TP. Evaluating quality management systems for HIV rapid testing services in primary healthcare clinics in rural KwaZulu-Natal, South Africa. PLOS ONE. 2017; 12(8): e0183044. doi: 10.1371/journal.pone.0183044 28829801 PMC5567898

[43] Yao K, Wafula W, Bile EC, Cheignsong R, Howard S, Demby A, et al. Ensuring the Quality of HIV Rapid Testing in Resource-Poor Countries Using a Systematic Approach to Training. American Journal of Clinical Pathology. 2010; 134(4): 568–572. doi: 10.1309/AJCPOPXR8MNTZ5PY 20855637

[44] Chiu YHC, Ong J, Walker S, Kumalawati J, Gartinah T, McPhee DA, et al. Photographed Rapid HIV Test Results Pilot Novel Quality Assessment and Training Schemes. PLOS ONE. 2011; 6(3): e18294. doi: 10.1371/journal.pone.0018294 21483842 PMC3069085

[45] Learmonth KM, McPhee DA, Jardine DK, Walker SK, Aye TT, Dax EM. Assessing proficiency of interpretation of rapid human immunodeficiency virus assays in nonlaboratory settings: Ensuring quality of testing. Journal of Clinical Microbiology. 2008; 46(5): 1692–1697. doi: 10.1128/JCM.01761-07 18353938 PMC2395071

[46] Kagulire SC, Opendi P, Stamper PD, Nakavuma JL, Mills LA, Makumbi F, et al. Field evaluation of five rapid diagnostic tests for screening of HIV-1 infections in rural Rakai, Uganda. International Journal of STD & AIDS. 2011; 22(6): 308–309. doi: 10.1258/ijsa.2009.009352 21680664 PMC3726838

[47] Bienek DR, Charlton DG. The Effect of Simulated Field Storage Conditions on the Accuracy of Rapid User-Friendly Blood Pathogen Detection Kits. Military Medicine. 2012; 177(5): 583–588. doi: 10.7205/milmed-d-11-00420 22645886

[48] Choko AT, Taegtmeyer M, MacPherson P, Cocker D, Khundi M, Thindwa D, et al. Initial Accuracy of HIV Rapid Test Kits Stored in Suboptimal Conditions and Validity of Delayed Reading of Oral Fluid Tests. PLOS ONE. 2016; 11(6): e0158107. doi: 10.1371/journal.pone.0158107 27336161 PMC4918937

[49] Facente SN, Dowling T, Vittinghoff E, Sykes DL, Colfax GN. False Positive Rate of Rapid Oral Fluid HIV Tests Increases as Kits Near Expiration Date. PLOS ONE. 2009; 4(12): e8217. doi: 10.1371/journal.pone.0008217 20011584 PMC2785875

