# Supplementary material for: Development of an approach to monitor the manufacturing consistency of HIV rapid diagnostic tests: Panel qualification and potential impact on country programs
Source: PLoS One. 2023 Apr 10;18(4):e0284175. doi: 10.1371/journal.pone.0284175 (PMC10085047; doi:10.1371/journal.pone.0284175)
Supplement: S1 Table — (DOCX) [file pone.0284175.s001.docx]

| Positive |  |  |  |  |  |  | Negative |  |  |  |  |  |
| --- | --- | --- | --- | --- | --- | --- | --- | --- | --- | --- | --- | --- |
| Specimen | Product A | Product B | Product C | Product D | Product E |  | Specimen | Product A | Product B | Product C | Product D | Product E |
| P001PM | NO | NO | NO | NO | NO |  | N001PM | YES | YES | YES | YES | YES |
| P002PM | YES | YES | YES | YES | YES |  | N002PM | YES | YES | YES | YES | YES |
| P003PM | YES | YES | YES | YES | YES |  | N003PM | YES | YES | YES | YES | YES |
| P004PM | YES | YES | YES | YES | YES |  | N004PM | YES | YES | YES | YES | YES |
| P005PM | YES | YES | YES | YES | YES |  | N005PM | YES | YES | YES | YES | YES |
| P006PM | YES | YES | YES | YES | YES |  | N006PM | YES | YES | YES | YES | YES |
| P007PM | YES | YES | YES | YES | YES |  | N007PM | YES | YES | YES | YES | YES |
| P008PM | YES | YES | YES | YES | YES |  | N008PM | YES | YES | YES | YES | YES |
| P009PM | YES | YES | YES | YES | YES |  | N009PM | YES | YES | YES | YES | YES |
| P010PM | YES | YES | YES | YES | YES |  | N010PM | YES | YES | YES | YES | YES |
| P011PM | YES | YES | YES | YES | YES |  | N011PM | YES | YES | YES | YES | YES |
| P012PM | YES | YES | YES | YES | YES |  | N012PM | YES | YES | YES | YES | YES |
| P013PM | YES | YES | YES | YES | YES |  | N013PM | YES | YES | YES | YES | YES |
| P014PM | YES | YES | YES | YES | YES |  | N014PM | YES | YES | YES | YES | YES |
| P015PM | YES | YES | YES | YES | YES |  | N015PM | YES | YES | YES | YES | YES |
| P016PM | YES | YES | YES | YES | YES |  | N016PM | YES | YES | YES | YES | YES |
| P017PM | YES | YES | YES | YES | YES |  | N017PM | YES | YES | YES | YES | YES |
| P018PM | YES | YES | YES | YES | YES |  | N018PM | YES | YES | YES | YES | YES |
| P019PM | YES | YES | YES | YES | YES |  | N019PM | YES | YES | YES | YES | YES |
| P020PM | YES | YES | YES | YES | YES |  | N020PM | YES | YES | YES | YES | YES |
| P021PM | YES | YES | YES | YES | YES |  | N021PM | YES | YES | YES | YES | YES |
| P022PM | YES | YES | YES | YES | YES |  | N022PM | YES | YES | YES | YES | YES |
| P023PM | YES | YES | YES | YES | YES |  | N023PM | YES | YES | YES | YES | YES |
| P024PM | YES | YES | YES | YES | YES |  | N024PM | YES | YES | YES | YES | YES |
| P025PM | YES | YES | YES | YES | YES |  | N025PM | YES | YES | YES | YES | YES |
| P026PM | YES | YES | YES | YES | YES |  | N026PM | YES | YES | YES | YES | YES |
| P027PM | YES | YES | NO | YES | YES |  | N027PM | YES | YES | YES | YES | YES |
| P028PM | YES | YES | NO | YES | YES |  | N028PM | YES | YES | YES | YES | YES |
| P029PM | YES | YES | NO | YES | YES |  | N029PM | YES | YES | YES | YES | YES |
| P030PM | YES | YES | YES | YES | YES |  | N030PM | YES | YES | YES | YES | YES |
| P031PM | YES | YES | YES | YES | YES |  | N031PM | YES | YES | YES | YES | YES |
| P032PM | YES | YES | YES | YES | YES |  | N032PM | YES | YES | YES | YES | YES |
| P033PM | YES | YES | YES | YES | YES |  | N033PM | YES | YES | YES | YES | YES |
| P034PM | YES | YES | YES | YES | YES |  | N034PM | YES | YES | YES | YES | YES |
| P035PM | YES | YES | YES | YES | YES |  | N035PM | YES | YES | YES | YES | YES |
| P036PM | YES | YES | NO | YES | YES |  | N036PM | YES | YES | YES | YES | YES |
| P037PM | YES | YES | YES | YES | YES |  | N037PM | YES | YES | YES | YES | YES |
| P038PM | YES | YES | NO | YES | YES |  | N038PM | YES | YES | YES | YES | YES |
| P039PM | YES | YES | YES | YES | YES |  | N039PM | YES | YES | YES | YES | YES |
| P040PM | YES | YES | YES | YES | YES |  | N040PM | YES | YES | YES | YES | YES |
| P041PM | YES | YES | YES | YES | YES |  | N041PM | YES | YES | YES | YES | YES |
| P042PM | NO | YES | NO | NO | YES |  | N042PM | YES | YES | YES | NO | YES |
| P043PM | YES | YES | YES | YES | YES |  | N043PM | YES | YES | YES | YES | YES |
| P044PM | YES | YES | YES | YES | YES |  | N044PM | YES | YES | YES | YES | YES |
| P045PM | YES | YES | YES | YES | YES |  | N045PM | YES | YES | YES | YES | YES |
| P046PM | YES | YES | YES | YES | YES |  | N046PM | YES | YES | YES | YES | NO |
| P047PM | YES | YES | YES | YES | YES |  | N047PM | YES | YES | YES | YES | YES |
| P048PM | YES | YES | YES | YES | YES |  | N048PM | YES | YES | YES | YES | YES |
| P049PM | YES | YES | YES | YES | YES |  | N049PM | YES | YES | YES | YES | YES |
| P050PM | YES | YES | YES | YES | YES |  | N050PM | YES | YES | YES | YES | YES |
| P051PM | YES | YES | YES | YES | YES |  | N051PM | YES | YES | YES | YES | YES |
| P052PM | YES | YES | YES | YES | YES |  | N052PM | YES | YES | YES | YES | YES |
| P053PM | YES | YES | YES | YES | YES |  | N053PM | YES | YES | YES | YES | YES |
| P054PM | YES | YES | YES | YES | YES |  | N054PM | YES | YES | YES | YES | YES |
| P055PM | YES | YES | YES | YES | YES |  | N055PM | YES | YES | YES | YES | YES |
| P056PM | YES | YES | YES | YES | YES |  | N056PM | YES | YES | YES | YES | YES |
| P057PM | YES | YES | YES | YES | YES |  | N057PM | YES | YES | YES | YES | YES |
| P058PM | YES | YES | YES | YES | YES |  | N058PM | YES | YES | YES | YES | YES |
| P059PM | YES | YES | YES | YES | YES |  | N059PM | YES | YES | YES | YES | YES |
| P060PM | YES | YES | YES | YES | YES |  | N060PM | YES | YES | YES | YES | YES |
| P061PM | YES | YES | YES | YES | YES |  | N061PM | YES | YES | YES | YES | YES |
| P062PM | YES | YES | YES | YES | YES |  | N062PM | YES | YES | YES | YES | YES |
| P063PM | YES | YES | YES | YES | YES |  | N063PM | YES | YES | YES | YES | YES |
| P064PM | YES | YES | YES | YES | YES |  | N064PM | YES | YES | YES | YES | YES |
| P065PM | YES | YES | NO | YES | YES |  | N065PM | YES | YES | YES | YES | YES |
| P066PM | YES | YES | YES | YES | YES |  | N066PM | YES | YES | YES | YES | YES |
| P067PM | YES | YES | YES | YES | YES |  | N067PM | YES | YES | YES | YES | YES |
| P068PM | YES | YES | YES | YES | YES |  | N068PM | YES | YES | YES | YES | YES |
| P069PM | YES | YES | YES | YES | YES |  | N069PM | YES | YES | YES | YES | YES |
| P070PM | YES | YES | YES | YES | YES |  | N070PM | YES | YES | YES | YES | YES |
| P071PM | YES | YES | YES | YES | YES |  | N071PM | YES | YES | YES | YES | YES |
| P072PM | YES | YES | YES | YES | YES |  | N072PM | YES | YES | YES | YES | YES |
| P073PM | YES | YES | YES | YES | YES |  | N073PM | YES | YES | YES | YES | YES |
| P074PM | YES | NO | NO | NO | NO |  | N074PM | YES | YES | YES | YES | YES |
| P075PM | YES | YES | YES | YES | YES |  | N075PM | YES | YES | YES | YES | YES |
| P076PM | YES | YES | YES | YES | YES |  | N076PM | YES | YES | YES | YES | YES |
| P077PM | YES | YES | YES | YES | YES |  | N077PM | YES | YES | YES | YES | YES |
| P078PM | YES | YES | YES | YES | YES |  | N078PM | YES | NO | YES | YES | YES |
| P079PM | YES | YES | YES | YES | YES |  | N079PM | YES | YES | YES | YES | YES |
| P080PM | YES | YES | YES | YES | YES |  | N080PM | YES | YES | YES | YES | YES |
| P081PM | YES | YES | YES | YES | YES |  | N081PM | YES | YES | YES | YES | YES |
| P082PM | YES | YES | YES | YES | YES |  | N082PM | YES | YES | YES | YES | YES |
| P083PM | YES | YES | YES | YES | YES |  | N083PM | YES | YES | YES | YES | YES |
|  |  |  |  |  |  |  | N084PM | YES | YES | YES | YES | YES |
